# Portable Nanocomposite System for Wound Healing in Space

**DOI:** 10.3390/nano13040741

**Published:** 2023-02-15

**Authors:** Chiara Zagni, Andrea Antonino Scamporrino, Paolo Maria Riccobene, Giuseppe Floresta, Vincenzo Patamia, Antonio Rescifina, Sabrina Carola Carroccio

**Affiliations:** 1 Department of Drug and Health Sciences, University of Catania, V.le A. Doria 6, 95125 Catania, Italy; 2 Institute for Polymers, Composites, and Biomaterials CNR-IPCB, Via Paolo Gaifami 18, 95126 Catania, Italy

**Keywords:** cryogel, wound dressing, synthesis, HEMA, HNTs, thymol

## Abstract

It is well known that skin wound healing could be severely impaired in space. In particular, the skin is the tissue at risk of injury, especially during human-crewed space missions. Here, we propose a hybrid system based on the biocompatible poly 2-hydroxyethyl methacrylate (pHEMA) to actively support a nanocontainer filled with the drug. Specifically, during the cryo-polymerization of HEMA, halloysite nanotubes (HNTs) embedded with thymol (Thy) were added as a component. Thy is a natural pharmaceutical ingredient used to confer wound healing properties to the material, whereas HNTs were used to entrap the Thy into the lumen to ensure a sustained release of the drug. The as-obtained material was characterized by chemical–physical methods, and tests were performed to assess its ability for a prolonged drug release. The results showed that the adopted synthetic procedure allows the formation of a super absorbent system with good swelling ability that can contain up to 5.5 mg of Thy in about 90 mg of dried sponge. Releasing tests demonstrated the excellent material’s ability to perform a slow controlled delivery of 62% of charged Thy within a week. As humans venture deeper into space, with more extended missions, limited medical capabilities, and a higher risk of skin wounds, the proposed device would be a versatile miniaturized device for skin repair in space.

## 1. Introduction

The human body’s capacity to be healed depends on several factors [1]. One of these is microgravity, which induces changes in mechanisms underlying tissue repair [2]. The long permanence of mice in orbit (3 months), corresponding to several years in human life [3], has proved how reducing gravity impacts mouse skin physiology by alternating the dermal, hair follicles, and muscular compartments of the skin [4]. Nowadays, space mission involves long-term space exploration that increases astronauts’ time in reduced gravity conditions [5]. This could inevitably expose the cosmonauts to injuries and traumatic events. The increasing number of deep space exploration missions, commercial spaceflights, and incoming space tourism could represent a significant concern for astronauts and people about cutaneous alterations and skin deterioration. Therefore, miniaturized disposal that could help in case of wound scratches should be a must in space [6,7].

In this view, wound dressing containing an active ingredient would be a great solution. In the last few years, using natural substances has become a source of new therapeutic discoveries for wound healing [8]. The monoterpene thymol (Thy), found in essential oils of the genus *Origanum*, presents antibacterial, antifungal [9], anti-inflammatory, and analgesic effects [10,11]. These properties make this compound a strong candidate for the development of products for tissue repair. Thy acts in the three phases by promoting re-epithelialization, angiogenesis, and the growth of fibroblasts and keratinocytes [12].

Wound dressings are commonly used in clinical practice to clean, cover, and protect the wound from the external environment [13]. They cover the injury, provide a temporary barrier against external infections and accelerate the healing process [14]. Hydrogel dressings are an emerging area for wound care, as they increase the speed and success of wound healing [15]. To date, the number of products produced by nanotechnology or containing nanomaterials is increasing. Current applications include healthcare (drug delivery, regenerative medicine, and diagnostics), environmental protection, electronics, cosmetics, and information technology [16,17,18,19,20,21,22].

Cryogels are macroporous hydrogels widely utilized in various biomedical applications, including wound dressings [23,24]. Compared to hydrogel, cryogels show superior mechanical and swelling properties thanks to their large and interconnected porosity [25]. Poly 2-hydroxyethyl methacrylate (pHEMA)-based cryogels were chosen to prepare antimicrobial cryogel wound dressings. A super porous cryogel composed of p(HEMA) and poly(tannic acid) (pTA) or lysozyme-imprinted poly (hydroxyethyl methacrylate-*N*-methacryloyl-(l)-histidine methyl ester) (pHEMA-MAH) cryogel membranes are only a few examples of HEMA-based cryogels for wound dressing with antimicrobial activity [26,27]. The common usage of p(HEMA) in cryogel wound dressings is an expected outcome as it rapidly forms gels and is already used for many biomedical applications. Recently, Zhang et al. developed an allochroic and zwitterionic polymer of (pVPES) as a wound dressing that can also be implanted to monitor changes during wound healing [28]. At the same time, Wan et al. have devised multifunctional biocompatible hydrogels based on acryloyl-6-aminocaproic acid (AA) and *N*-acryloyl 2-glycine (NAG) coordinating Ca^2+^. AA/NAG/Ca hydrogels, thanks to their excellent mechanical, adhesion, self-healing, and hemostatic properties, could be applied to manage postoperative wounds and prevent postoperative tissue adhesions [29]. Poly-*N*-(2-Hydroxyethyl)acrylamide added with Osteichthyes-extracted gelatin was also developed as a novel strategy to obtain a sticky hydrogel for dynamic adhesive dressings and wound healing. Gelatin/PHEAA hydrogel contained essential tissue growth factors that promoted blood coagulation, also exhibiting excellent hemostatic properties [30].

Halloysite is a new green and naturally abundant nanomaterial, an inexpensive nanoscale container for encapsulating biologically active molecules [31]. Natural halloysite (Al_2_Si_2_O_5_(OH)_4_·nH_2_O) nanotubes (HNTs) are clay materials with hollow tubular structures in the nanometer range [32]. They usually have an internal diameter of 10–30 nm, an external diameter of 40–70 nm, and a length between 200 and 2000 nm [33]. HNTs’ internal surface consists of Al-OH groups, while the outer shell comprises siloxane groups (Si-O-Si) [34]. The inner lumen can accommodate drugs, DNA, or proteins, while the wall interlayer spaces can be filled with small drug molecules. Moreover, the molecules can be adsorbed (or covalently bound) on the surface [35]. These different interactions resulted in an initial drug burst release starting from the outer surface of the tubes and followed by a sustained release due to the slower leakage of the drug from the tube’s interior lumens [36,37].

To have a portable drug container for skin scratches with sustained release properties and very tiny dimensions, we propose combining HEMA cryogel with HNTs as a nanocontainer to load Thy. The novel system consists of a patch that can be easily applied to the skin, allowing the controlled release of the anti-inflammatory and antibacterial natural compound Thy to accelerate the wound healing process.

## 2. Materials and Methods

### 2.1. Materials

HEMA, *N*,*N*′-methylene-bisacrylamide (MBAA), ammonium persulfate (APS), tetramethyl-ethylene-diamine (TEMED), HNTs, and Thy were all purchased from Sigma-Aldrich and used as received. A Milli-Q water purification system produced deionized water.

### 2.2. Synthesis of Cryogels

#### 2.2.1. Preparation of HNTs/Thy Nanohybrid

An amount of 3 g of Thy was dispersed in 10 mL of Millipore water at 50 °C for about 30 min. Then, 3 g of HNTs was added, and the resulting suspension solution was ultrasonicated, reaching a good HNTs dispersion. After that, it was heated at 50 °C for 3 min and subjected to a vacuum step for about 15 min. The solution was then shaken for 5 min, and at least three vacuum steps were performed. The hybrid Thy-nano-containers formed were dried at 50 °C to reach a stable weight. Thy content was calculated using Equation (1) [38]:

(1) $$\alpha_{3}=w\alpha_{1}+\left(1-w\right)\alpha_{2}$$

where *α*_1_ is the mass loss of Thy at 800 °C; *α*_2_ is the mass loss of HNTs at 800 °C; *α*_3_ is the mass loss of HNTs-Thy at 800 °C; and w is weight % of Thy calculated by using mass loss data obtained by TGA analysis. Thy content (wt%) in the HNTs-Thy hybrid was calculated to be 55.5%, and the HNTs content was 44.5%.

#### 2.2.2. Synthesis of HEMA and HEMA-HNT Cryogels

Here, 340 mg of HEMA (2.6 mmol) and 64 mg of crosslinker agent *N*,*N*′-methylene bisacrylamide MBAA (0.44 mmol) were added to 3.3 mL of deionized water and mixed until complete dissolution. The mixture was cooled at 0 °C, and then 1% *v/v* of a water solution of APS (at a concentration of 10% *w*/*v*) and 1% *v/v* of a water solution of TEMED (at a concentration of 10% *w*/*v*) were added. The total content of polymerizable compounds was ~10% *w/v* of the solution. The final mixture was slowly stirred and transferred into propylene syringes [39]. The cryo-polymerization reaction was conducted at −15 °C for 24 h. After that time, the frozen cryogels were thawed and washed several times with water. Finally, the samples were dried in a freeze-dryer. The polymerization yield was 89%.

HEMA-HNT cryogels were synthesized as previously described for HEMA by adding 10 mg of HNT to the monomer and crosslinker solution.

#### 2.2.3. Synthesis of HEMA-HNT/Thy Cryogel

For the synthesis of cryogel containing HNTs/Thy, we proceeded as previously described with the addition of 15 mg of the nanohybrid HNTs loaded with Thy.

**Figure 1:**
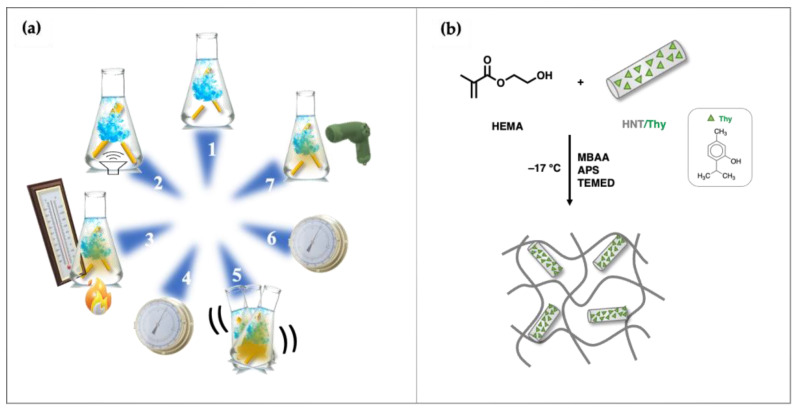
Schematic representation of (**a**) HTNs/Thy preparation: (1) Dissolution; (2) Mix; (3) Heating; (4) Vacuum; (5) Shaking; (6) Vacuum; (7) Drying. (**b**) Cryogel synthesis.

A diagram illustrating the structure and preparation process of HEMA and HEMA HNTs/Thy cryogels is presented in Figure 1.

### 2.3. Cryogels Characterization

The synthesized cryogels were characterized by various techniques to determine their physicochemical properties.

Fourier-transform infrared spectroscopy (FTIR) was used to determine the functional groups of the cryogels. Fourier-transform spectra were recorded in the 4000–400 cm^−1^ region using FTIR System 2000 (Perkin-Elmer, Waltham, MA, USA) and KBr as media.

The thermal stability of the samples was investigated by thermogravimetric analysis (TGA) using a thermogravimetric apparatus (TA Instruments Q500) under a nitrogen atmosphere (flow rate 60 mL/min) and a heating rate of 10 °C/min, from 50 °C to 800 °C. Samples were cut into different sections and weighed (*ca* 6 mg). The weight loss percentage was determined by TGA.

Scanning electron microscopy (SEM) Phenomenex microscope was used to study the morphology of the macroporous gels. Dried samples were cut into thin discs (1–2 mm thick) and sputtered with gold (<10 nm) to confer conductivity. The data were acquired and processed using Phenom Porometric 1.1.2.0 (Phenom-World BV, Eindhoven, The Netherlands).

Energy-dispersive X-ray spectroscopy (EDX) was used to obtain an analysis of the chemical elements of all prepared materials and determine their chemical composition.

Swelling tests were performed to estimate the water uptake capability of prepared sponges [39,40]. Small pieces of 9 mm diameter and 10 mm length were placed in deionized water for 24 h. Before weighing, each sample was taken out from the water, and the surface water was blotted. Each sample was weighed three times. The standard deviation was less than 5%.

### 2.4. Thymol Release

HEMA-HNT/Thy nanohybrid cryogels have been tested for their ability to release Thy in saline buffer consisting of a solution of PBS (Phosphate-Buffered Saline, pH 7.4).

A weighed sample piece was placed in a vial containing 10 mL of saline solution. Aliquots were collected at different times (5, 15, 30, 45, 90, 120, 140 min, 24, 48, 72, 94, 120, 144, and 168 h). The released amount of the drug was determined by a UV-Vis JASCO spectrophotometer using a wavelength range of 255–400 nm. The cuvette used has a volume of 4 mL and a path length of 1 cm. Each sample collected from the solution was diluted 8×.

Each measurement was performed in triplicate.

Drug release efficiency (*DRE*) was calculated from Equation (2):

(2) $$DRE\left(\%\right)=\frac{\text{Mass released drug}}{\text{Mass loaded drug}}\times 100$$

### 2.5. Kinetic of Release

The release behavior of Thy from HEMA-HNT/Thy cryogel has been compared with the most used mathematical models in drug delivery: zero order, first order, Higuchi, Weibull, and Korsmeyer–Peppas models were fitted to our data, and analysis of regression was performed [41].

Data used for the fitting process includes records obtained within 3 h of release; this choice was made to eliminate the interference between the saturation of the medium and the real kinetic behavior.

### 2.6. Statistical Analysis

All the experiments were performed in triplicate. Statistical analysis of the results expressed as mean ± standard deviation was performed using GraphPad Prism 9.0 (GraphPad Software, San Diego, CA, USA) and assessed using ANOVA and the Student’s *t*-test with a 95% significance level (*p* < 0.05). All relative errors considered in the experimental results were lower than 5%.

## 3. Results and Discussion

Due to their peculiar properties, hydrogels are already well-known materials for wound healing applications [42]. However, to maintain a better-oxygenated environment, favor a more controlled and sustained release of the drug, and gain material with improved mechanical properties, in this work, we have focalized our attention on cryogel, performing a standard polymerization procedure at a temperature below the freezing point of polymerization solvent [21,22,23]. This procedure allows us to obtain interconnected macroporous materials with excelled swelling ability, ideal for the goal to be applied to wounded skin and release the appropriate amount of the desired drug [24,25,26,27]. Cryogels are already tested as wound dressings; however, the main inconvenience of using these systems is imputable to an initial burst effect, which means a higher liberation of the drugs in the first minutes after the application of the patch. Moreover, cryogels are hydrophilic systems that can incorporate into their matrix hydrophilic drugs. Additionally, the mechanical properties of cryogels should be improved without thwarting the materials’ swelling and release properties. This is a difficult task since we need to increase the crosslinking to enhance mechanical properties, which inevitably reduces the abovementioned feature.

Thy is a colorless crystalline monoterpene phenol endowed with excellent pharmacological properties, including antioxidant, anti-inflammatory, analgesic, antibacterial, antifungal, and antiseptic activities [43]. The hydrophobic nature of Thy could represent a limitation for cryogels loading.

Therefore, to obtain a material able to load and release Thy and increase the system’s mechanical properties, we prepared a hybrid compound composed of halloysite nanotubes dispersed in the cryogel matrix. Halloysite and Thy can form several hydrogen bonds that efficiently favor the entrapping of Thy in the lumen. This procedure facilitates the incorporation of the drug into the nanotubes, exploiting at the same time the peculiarity of the cryogel to swell and gradually release the drug.

To entrap hydrophilic molecules, halloysite nanotubes were mixed with a saturated Thy solution in water and then exposed to a high vacuum [38]. Then, the nanotubes loaded with Thy were dispersed in a solution containing HEMA monomers and MBAA as the crosslinker. A free radical redox polymerization at −15 °C was used to form cryogels (Figure 1). In that way, the solvent freezes down, and the polymerization process occurs around the ice crystal. The thawing of the solvent leaves an interconnected macroporous structure. The size and shape of the pores are influenced mainly by the type and concentration of monomers and crosslinkers, the rate of solvent crystallization, the temperature and duration of cryogelation, and the cooling rate [24,25,26,27]. Figure 1 presents the synthesis and digital photographs of HEMA-HNT/Thy nanocomposite cryogel.

**Figure 2:**
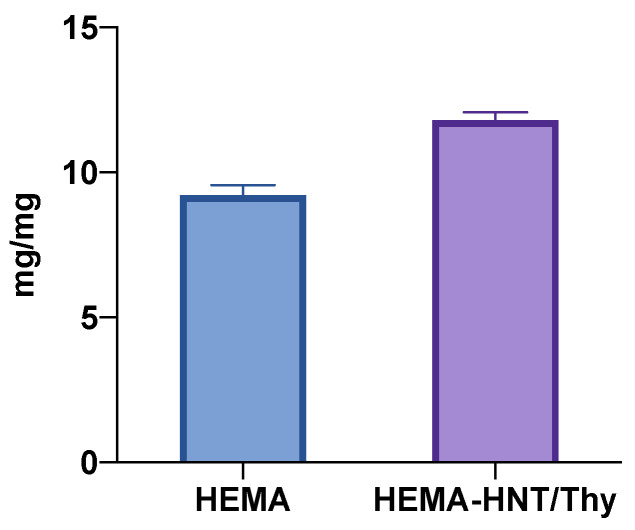
Swelling capacity of HEMA and HEMA-HNT/Thy cryogels.

The swelling properties of the nanohybrid and HEMA cryogels have been studied since the value of such properties is strictly correlated to drug release (Figure 2). The results showed that HEMA cryogel had a significantly lower swelling ability than HEMA_HNT/Thy cryogel. This is most likely due to the presence of both HNTs and Thy within the same hydrogels. Indeed, HNT may provide physical barriers to crosslinking reactions, whereas Thy could act as a stabilizer of radical propagation. In both cases, the minor numbers of chain connections imply the formation of a softer gel compared to the HNTs-free sample. Nevertheless, at the temperature used to form the cryogel (–15 °C), Thy is poorly soluble in water and entrapped into the HNT, so any reactions of it with other species present in the solution are strongly inhibited. The evidence of Thy presence after the reaction was confirmed by the UV-Vis profile that perfectly overlaps the ones of the pure Thy.

It is worth noticing that the increase in cryogel swelling can favor the burst effect, as wanted.

**Figure 3:**
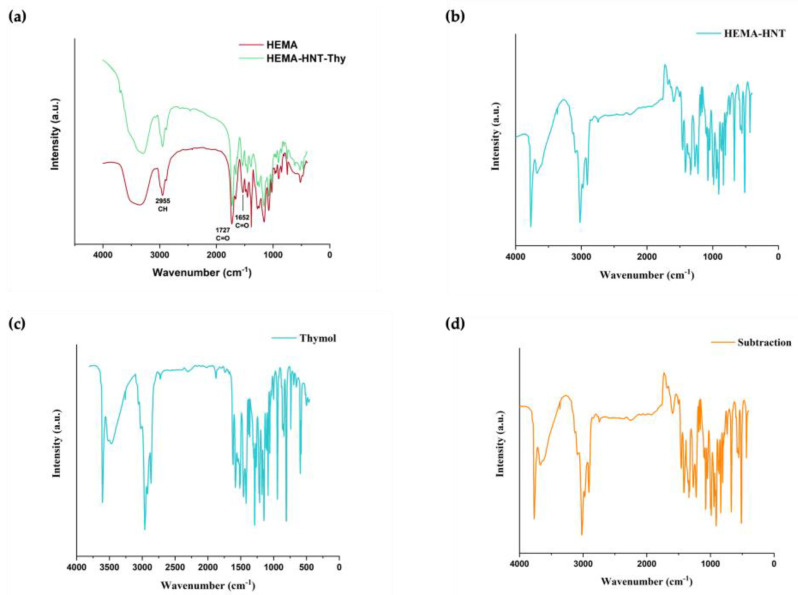
FTIR spectra of (**a**) HEMA and HEMA-HNT/Thy; (**b**) HEMA-HNT; (**c**) Thy; (**d**) HEMA-HNT/Thy with HEMA-HNT subtraction.

To confirm the synthesized material’s chemical composition, FTIR spectra were recorded. In Figure 3, spectra of HEMA, HEMA-HNT, and HEMA-HNT/Thy are reported.

The spectra show the characteristic peaks of HEMA crosslinked with MBAA in the 3500–3400 cm^−1^, corresponding to NH and OH stretching vibrations. It is also possible to notice the presence of CH stretching vibrations at 2955 cm^−1^. The peak at 1727 cm^−1^ was assigned to the ester stretching, whereas the signal at 1652 cm^−1^ was assigned to the amide crosslinker.

Specific absorption bands at 3695 cm^−1^ and 3621 cm^−1^ due to Al–O–OH bending and O–H stretching vibration confirm the presence of HNT. Moreover, the characteristic band of HNTs at 910 cm^−1^ is likely due to the deformation vibration of O–H in the inner-surface hydroxyl groups of Al–O–H the above hydroxyl groups.

More importantly, the disappearance of the peaks at 1637, 933, and 816 cm^−1^, corresponding to the typical signal of methylene (-C=C-) in the spectrum of HEMA monomer (not reported), are not present in the polymer spectrum, confirming the formation of the polymeric sponge.

Thy spectra (Figure 3c) show a band at 3229 cm^−1^ corresponding to phenolic OH stretching containing hydrogen bonding, while C=C stretching of the benzene ring stands at 1620 cm^−1^.

**Figure 4:**
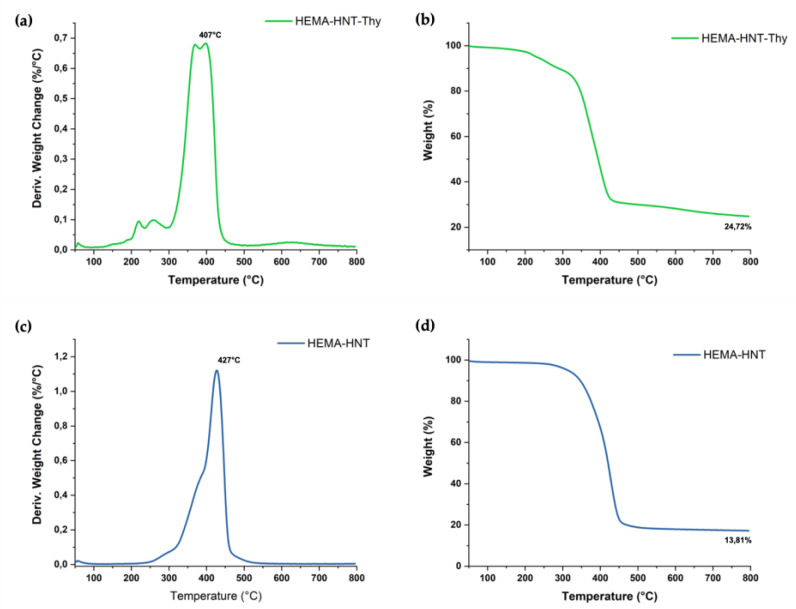
Thermogravimetrical curves TG and DTG of (**a**,**b**) HEMA-HNT/Thy (green) and (**c**,**d**) HEMA-HNT (blue).

The thermogravimetric analysis (TGA) of the new synthesized cryogel HEMA-HNT/Thy cryogel was performed (Figure 4) to assess the thermal property and the quantification of HNT content. As a reference, HEMA-HNT cryogel was synthesized using a similar procedure and analyzed. TG profiles in Figure 4a,b indicated that the amount of HNT filled in both samples ranges from 14 to 20% in weight. Compared to a sample containing only HNT, the HEMA-HNT/Thy cryogels showed the activation of several degradation weight loss steps. Specifically, as Shemesh et al. reported [34], the decomposition step picked at 220 °C is ascribable to the Thy, further confirming its presence into the formulation, and specifically embedded into the HNTs lumen. As reported in the literature [36,44,45], HNTs protect the organic molecule from the thermal process, preventing its decomposition at a lower temperature (160 °C for free Thy). As evidenced by TGA, the presence of Thy negatively influences the thermal stability of the cryogel since the hybrid material experienced a reduction in decomposition temperature from 427 °C to 407 °C. This result is not surprising if we consider that the Thy can work as a radical scavenger, decreasing the number of radical crosslinking reactions and, thus, the thermal stability of the sample. It is worth noticing that the depletion of thermal properties of HEMA-HNT/Thy does not affect the final application of the cryogel.

**Figure 5:**
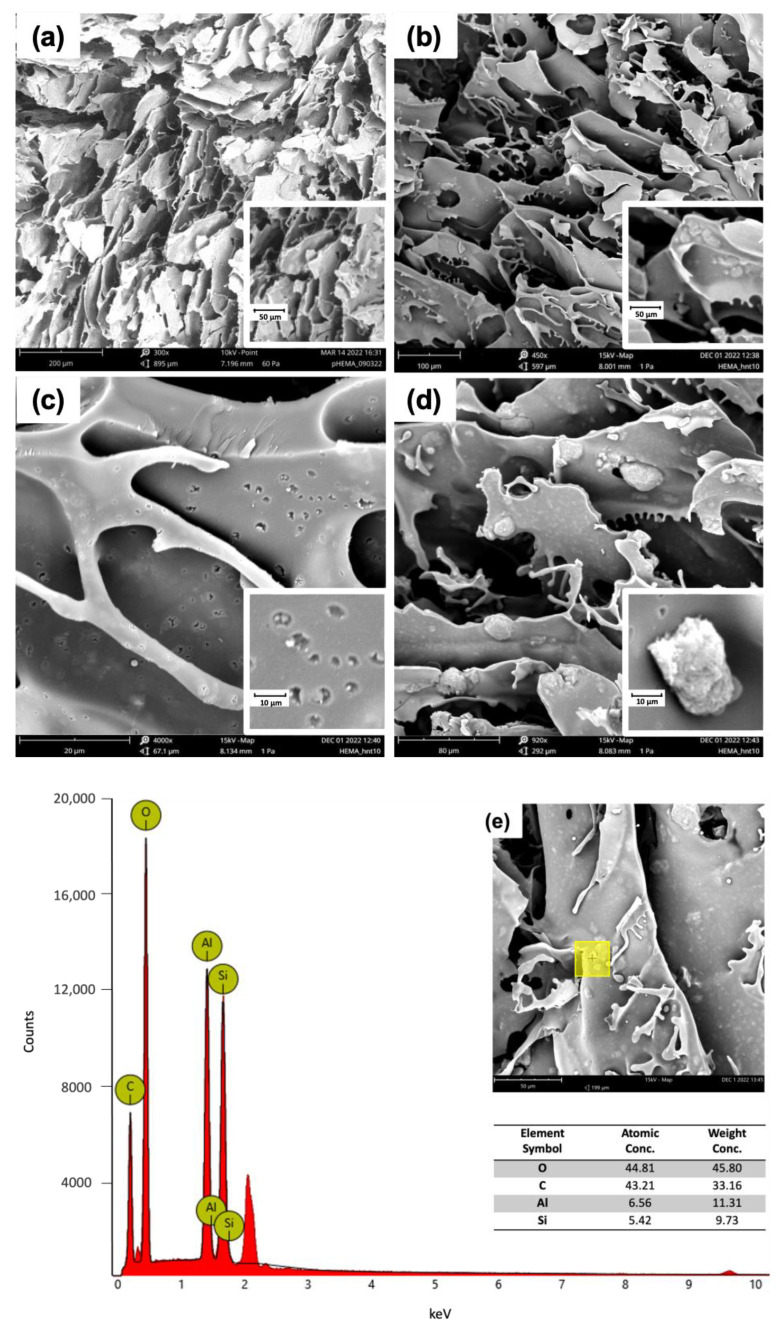
SEM images: (**a**) Cryogel of HEMA; (**b**) Cryogel of HEMA-HNT/Thy; (**c**) halloysite nanotubes inclusions; (**d**) halloysite aggregates. (**e**) EDX analysis of a portion (yellow square) of HEMA-HNT/Thy cryogel. The unlabeled peaks at 2.1 and 9.6 keV correspond to gold, which is used to confer conductivity.

SEM images (Figure 5) showed the characteristic macroporous structure of HEMA cryogel with a pore size ranging from 10 to 100 μm. In addition, HEMA-HNT/Thy cryogel reveals tiny pores with a diameter of 1–2 μm that, most likely, are formed thanks to the insertion of halloysite nanotubes into the polymer matrix during the synthesis. Moreover, EDX analysis confirmed the presence of the typical halloysite elements such as Al and Si (Figure 5).

**Figure 6:**
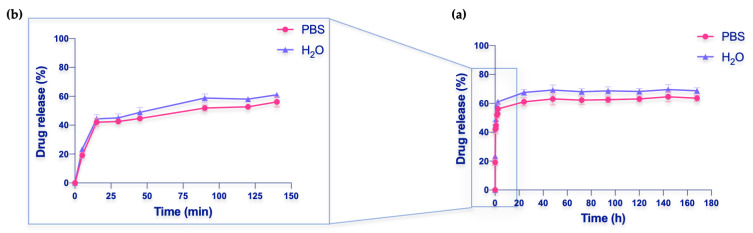
(**a**) Thy release (%) from HEMA-HNT/Thy in one week. (**b**) Enlargement of Thy release (%) in the first 140 min.

The release of Thy form HEMA-HNT nanocomposite cryogel was investigated in vitro by placing a piece of cryogel in both PBS saline buffer at pH = 7.4 and water. The amount of drug released was calculated by UV-Vis, measuring the absorbance values of the solution at different times (Figure 6). The collected data are plotted as the ratio of Thy concentration in the release medium (PBS solution or water) at a given time to the initial drug concentration in the cryogel (C/C_tot_). As observed, the drug is released gradually over time, displaying an initial burst effect, and maintaining a steady release for up to 7 days. As usually happens, drugs loaded into cryogels show an initial rapid burst release followed by slower drug release, as reported in the literature [46,47]. This was due to the variation of concentration that represents the driving force to the migration of the drug from the carrier to the water or PBS solutions. After 24 h, equilibrium was reached, with the Thy release (desorption) gaining 70% in water and 65% in PBS. As a result, HEMA cryogel was shown to be a good candidate for HNT/Thy loading and controlled release.

The observed kinetics of release well-fit with the aimed application. In addition, an initial burst effect is desired in the presence of a wound. Then, a sustained and prolonged drug release is requested to promote healing. The kinetics of the burst release of the drug, in perfect sink conditions, can be described as initial dissolution followed by a fast diffusion from clusters to the contiguous media of overlapping drug particles deposited on the surface [48]. The burst release is principally produced by the drug adsorbed in the pores on the surface of cryogels during the loading process [49].

The drug release profile was analyzed by fitting our data to the most common mathematical model used in drug delivery: zero order, first order, Higuchi, Weibull, and Korsmeyer–Peppas models [50]; the specific formulas are reported in Equations (3)–(7), respectively.

Zero-order release model:

(3) $$M_{t}/M_{\infty }=k_{0}t$$

First-order release model:

(4) $$M_{t}/M_{\infty }=1-e^{-k_{1}t}$$

Higuchi model:

(5) $$M_{t}/M_{\infty }=k_{2}t$$

Weibull model:

(6) $$\frac{M_{t}}{M_{\infty }}=1-\exp\left(-at^{b}\right)$$

Peppas model:

(7) $$M_{t}/M_{\infty }=k_{3}t^{n}$$

**Figure 7:**
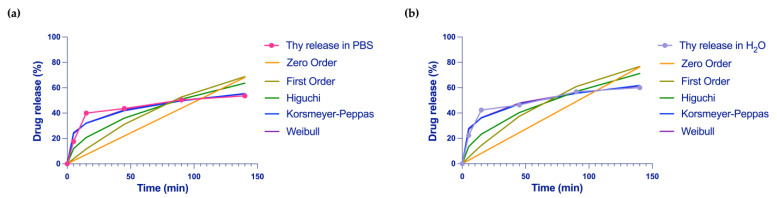
Kinetic release fitting for the Thy from HEMA-HNT/Thy cryogel in (**a**) saline buffer and (**b**) water.

As a result of the fitting analysis, Weibull and Korsmeyer–Peppas models showed the highest score (Figure 7).

The best fitted among the equations evaluated, illustrating drug release pattern from HEMA-HNT/Thy cryogel, resulted from the Weibull model. This model shows an R^2^ value ranging from a minimum of 0.95 for the saline buffer and 0.96 for the water to a maximum of 0.97 and 0.99, respectively (Table 1). The Weibull model was followed by the Korsmeyer–Peppas model that exhibits an R^2^ value ranging from a minimum of 0.97 to a maximum of 0.98.

The Korsmeyer–Peppas model originated from Fick’s law theory and is an ideal drug-release kinetic model [51]. In the Peppas model, the diffusional exponent, *n*, is an important indicator of the mechanism of transport of a drug through the polymer [52]. When *n* is less than 0.45, the drug release mechanism belongs to Fick diffusion, and the release is mainly based on drug diffusion. From our fitting results, *n* has a value of 0.24, which is less than 0.45, indicating that the sustained release of Thy belongs to Fick diffusion, and the drug dissolution is dominated by diffusion. In our release experiments, the key factor of forcing Thy’s rapid desorption and dissolution from the polymeric cryogel is the hydration of the matrix.

These results revealed that the drug release phenomenon for nanohybrid cryogel is prevalently polymer dependent rather than drug dependent. Considering the best-fitting model, we can conclude that the release consists of a two-stage model. Initially, the medium enters the matrix, causing the swelling of the cryogel. As a consequence, the leaching of the drug placed in the external HNTs surface occurs. Along with the time, HNTs cavity gradually loses the drug molecules filled into the lumen by ensuring a sustainable release. The burst effect of our system is well described by Weibull and Korsmeyer–Peppas models reflecting the presence of a two-stage model process for drug release of our cryogels as seen in analog materials.

## 4. Conclusions

This work proposed the synthesis of new HEMA-HNT/Thy-based crosslinked cryogels containing Thy using the cryopolymerization technique. This method allowed us to obtain a macroporous system with different features. The system has been developed to serve as a portable wound dressing to protect injured skin, especially during a space mission. The green active principle Thy was incorporated into the halloysite lumen of the wound dressings to enhance healing and prevent microbial infection. The swelling test confirmed the ability of the system to entrap water, allowing the absorption of the exudate, which is vital to maintain a clean environment for healing. The system has been tested for its ability to release Thy, simulating the in vivo environment. The release of the compound useful in wound healing was sustained and reached a value of 62%, exhibiting a bust effect and maintaining a constant release for one week. The kinetic release data show the typical pattern of a macroporous system perfectly fitting the Weibull model.

In conclusion, this new disposal, prepared as a portable system for wound healing, could be charged with different drugs and used for several purposes.

## Figures and Tables

**Table 1:** Kinetic release parameters for Thy from cryogel samples in PBS and H_2_O.

Thy Release													
Sample	Media	Zero Order		First Order		Higuchi		Korsmeyer–Peppas			Weibull		
		K	R^2^	K	R^2^	K	R^2^	K	n	R^2^	a	b	R^2^
Hema-HNT/Thy	PBS	0.487	0.795	0.008	0.850	5.375	0.918	16.523	0.245	0.973	0.161	0.324	0.976
Hema-HNT/Thy	H_2_O	0.545	0.808	0.010	0.873	6.014	0.929	18.877	0.240	0.986	0.184	0.333	0.988

## Data Availability

Not applicable.
